# Short-Term Outcomes of Impella-Supported High-Risk Percutaneous Coronary Intervention in Surgically Ineligible Patients: Insights From PROTECT III

**DOI:** 10.1016/j.jscai.2025.103859

**Published:** 2025-08-26

**Authors:** Batla Falah, Behnam N. Tehrani, Julia B. Thompson, Yousuf Shah, Yiran Zhang, Tayyab Shah, Michael J. Schonning, Arsalan Abu-Much, David J. Cohen, Björn Redfors, Deepak Acharya, Samer M. Garas, Suzanne J. Baron, Mir B. Basir, Aditya S. Bharadwaj, Cindy L. Grines, Alejandro Lemor, Alexander G. Truesdell, William W. O’Neill, Wayne B. Batchelor

**Affiliations:** a Clinical Trials Center, Cardiovascular Research Foundation, New York, New York; b Interventional Cardiology Department, Inova Schar Heart and Vascular, Inova Fairfax Hospital, Falls Church, Virginia; c School of Medicine and Dentistry, University of Rochester, Rochester, New York; d Division of Cardiovascular Medicine, Penn Medicine, University of Pennsylvania Health System, Philadelphia, Pennsylvania; e Division of Cardiology, Department of Internal Medicine, Yale School of Medicine, New Haven, Connecticut; f Department of Cardiology, St. Francis Hospital, Roslyn, New York; g Department of Population Health Sciences, Weill Cornell Medicine, New York, New York; h Department of Molecular and Clinical Medicine, Gothenburg University, Gothenburg, Sweden; i Department of Cardiology, Sahlgrenska University Hospital, Gothenburg, Sweden; j Sarver Heart Center, University of Arizona College of Medicine, Tucson, Arizona; k Department of Cardiology, Ascension St. Vincent, Jacksonville, Florida; l Massachusetts General Hospital, Boston, Massachusetts; m Baim Institute for Clinical Research, Boston, Massachusetts; n Center for Structural Heart Disease, Division of Cardiology, Henry Ford Health System, Detroit, Michigan; o Division of Cardiology, Loma Linda University Medical Center, Loma Linda, California; p Department of Cardiology, Northside Hospital Heart Institute, Atlanta, Georgia; q Department of Cardiology, University of Mississippi Medical Center, Jackson, Mississippi; r Virginia Heart, Falls Church, Virginia

**Keywords:** coronary artery bypass graft surgery, high-risk percutaneous coronary intervention, major adverse cardiovascular and cerebrovascular events, mechanical circulatory support, percutaneous left ventricular assist device

## Abstract

**Background:**

Impella-supported high-risk percutaneous coronary intervention (HRPCI) is an alternative for patients ineligible for coronary artery bypass grafting (CABG). However, limited data exist on patient characteristics, reasons for surgical turndown, and patient outcomes. This study aimed to characterize the baseline characteristics and short-term and intermediate-term outcomes of patients evaluated for CABG in the PROTECT III study.

**Methods:**

Patients enrolled in the PROTECT III study (NCT04136392), who underwent Impella-supported HRPCI, with an evaluable chart who were assessed by a cardiothoracic surgeon (CTS) for CABG were studied. Reasons for surgical turndown were derived from medical records. Baseline characteristics and major adverse cardiovascular and cerebrovascular events (composite of all-cause death, myocardial infarction, stroke/transient ischemic attack, and repeat revascularization) at 30 and 90 days and all-cause mortality at 1 year were assessed. Observed to expected 30-day mortality ratios were calculated using the Society of Thoracic Surgeons (STS) risk score.

**Results:**

Of 791 patients evaluated for CABG, 680 (86.0%) were turned down by a CTS, and 111 (14.0%) declined surgery. The most common reasons for surgical turndown were comorbidities (40%) and anatomical factors (25%). Compared with patients who declined surgery, patients turned down (deemed ineligible) by CTS had higher rates of major adverse cardiovascular and cerebrovascular event at 30 days (9.2% vs 4.6%; *P* = .12) and 90 days (14.1% vs 4.6%; *P* = .02). The observed to expected mortality ratio, based on the STS risk score, was 1.43 (95% CI, 1.08-1.83).

**Conclusions:**

Impella-supported HRPCI is a viable alternative for high-risk patients deemed ineligible for CABG. Patients turned down by a CTS had worse clinical outcomes than those who declined surgery. The underestimation of 30-day mortality by the STS risk score suggests the need for improved risk prediction models in this high-risk cohort.

## Introduction

Complex coronary artery disease (CAD), including left main and multivessel disease, affects nearly half of patients presenting with acute coronary syndrome.^1^^,^^2^ Selecting the optimal revascularization strategy in this setting may be challenging and is influenced by multiple factors, including patient age, frailty, presence of significant medical comorbidities, extent of CAD, complex lesion characteristics (eg, calcification, thrombus, long lesion length, total occlusion, and bifurcation anatomy), left ventricular (LV) systolic function, hemodynamic stability, and input from a multidisciplinary heart team.^3^^,^^4^ Several randomized clinical trials have shown that coronary artery bypass graft surgery (CABG) offers superior long-term outcomes compared with percutaneous coronary intervention (PCI) in patients with extensive multivessel disease.^5^, ^6^, ^7^, ^8^ Consequently, current guidelines favor the former as the preferred revascularization strategy for patients with complex CAD.^9^^,^^10^ Despite this, a notable proportion of patients deemed eligible for CABG either decline surgery or are deemed ineligible by a cardiothoracic surgeon (CTS). A recent study of 4824 patients deemed anatomically and medically suitable for CABG by a heart team showed that only 59% proceeded with surgery, while 33% of patients declined surgery, and another 8% were ultimately deemed ineligible by a CTS.^11^ As the likelihood of surgical ineligibility increases with operative risk, higher risk patients for CABG are often referred for high-risk complex PCI, which may require temporary hemodynamic support to mitigate the inherent risk of hemodynamic instability in these procedures.^12^

Although recent studies have demonstrated favorable outcomes for surgically ineligible patients who undergo high-risk percutaneous coronary intervention (HRPCI),^13^^,^^14^ there is a paucity of data on such patients who receive temporary mechanical circulatory support for their procedure. This study aimed to compare baseline characteristics and short-term outcomes of patients who underwent HRPCI in the cVAD PROTECT III percutaneous left ventricular assist device (pLVAD) registry who were evaluated by a CTS and turned down vs those of patients who declined.

## Materials and methods

### Study design, population, and oversight

The PROTECT III study design has been described previously^15^ (The Global cVAD Study [cVAD]; ClinicalTrials.gov: NCT04136392). Briefly, PROTECT III is an FDA-audited, single-arm, observational study that prospectively enrolled 1237 patients across 46 centers in North America between March 2017 and March 2020. Enrolled patients in the PROTECT III study underwent elective or urgent HRPCI procedures (ie, without cardiogenic shock) with an Impella 2.5 or Impella CP pLVAD implanted for procedural hemodynamic support. The decision to use an Impella device and the indications for HRPCI were left to physician discretion and the local standard of care. Patients met enrollment criteria once a decision was made to use Impella before or during the index PCI. Bailout pLVAD implantation after PCI was excluded.

Baseline patient demographic characteristics, echocardiographic data, and procedural characteristics were collected before the index procedure. Angiographic data were analyzed by an independent core laboratory (Beth Israel Deaconess Medical Center Angiographic Core Laboratory). All clinical events were adjudicated by an independent Clinical Events Committee through 90 days and patients were followed up to 1 year for vital status assessment.

### Ethical statement

The sponsor (Abiomed) oversaw study data management and source document verification and provided funding to the Cardiovascular Research Foundation and to Meducator for independent statistical analysis. The authors had unrestricted access to the study data and accept responsibility for the integrity of this report. The study was conducted in accordance with the Declaration of Helsinki and approved by each site’s institutional review board or independent ethics committee before enrollment.

### Study population

Patients with an evaluable chart who were enrolled in the PROTECT III study and evaluated by a CTS for CABG eligibility were included in this study. The primary reason for undergoing Impella pLVAD-supported HRPCI vs CABG was ascertained using chart review by an author (B.F.).

### Study end points

The primary end point was 90-day major adverse cardiovascular and cerebrovascular events (MACCE), defined as the composite of all-cause mortality, myocardial infarction, stroke/transient ischemic attack, and repeat revascularization. Secondary end points included 1-year all-cause mortality and 30-day MACCE. In-hospital adverse events (AEs; eg, pericardiocentesis, cardiac arrest, cardiogenic shock, major ventricular arrhythmia, acute kidney injury of ≥stage 2, life-threatening/disabling/major bleeding [Bleeding Academic Research Consortium scale of ≥3a], anemia requiring blood transfusion, prolonged intubation, vascular or cardiac complication requiring surgery or reintervention, and stroke/transient ischemic attack) and PCI-related complications (no reflow, abrupt vessel closure, coronary dissection, distal embolization, and perforation) were also assessed. Detailed definitions of AEs in the PROTECT III study have been published previously.^15^

### Society of Thoracic Surgeons risk calculation

The Society of Thoracic Surgeons (STS) predicted risk of 30-day mortality was calculated using the online STS Operative Risk Calculator (version 2.0.5). A Python script (version 3.9) was created that automated the input of each subject’s data into the online risk calculator. The script operated by mapping the assigned variable to the correct location in the calculator for each subject. If information regarding a particular covariate was not available for a specific patient, it was left blank. Fifty patient entries were confirmed manually to validate the accuracy of the methodology used by the Python script.

### Statistical analyses

Baseline characteristics were summarized with mean ± SD or median and IQR for continuous measures and proportions for categorical variables. For comparisons between the study groups, categorical variables were compared using the χ^2^ or Fisher exact test, while continuous variables were compared using ANOVA and Wilcoxon rank-sum test. For time-to-first event analyses, event rates were estimated by the Kaplan-Meier method and compared with the log-rank test. Event rates were compared between patients turned down by a CTS and those who declined to undergo surgery.

The observed 30-day AEs (stroke, reoperation, renal failure, and mortality) in the cVAD PROTECT III patient cohort were compared with the corresponding event rate predicted by the STS risk score (expected) in 2 ways. First, the ratio of observed to expected event rate was calculated for each outcome along with its 95% CI. Second, we examined the relationship between the observed and expected mortality by dividing patients into deciles of STS-predicted mortality and plotting observed vs predicted mortality rates for each decile. Goodness of fit was tested by using *R*^2^ values, and the line of best fit was compared with the line of identity using an *F* test for the null hypothesis that the slope is 1, and a *t* test for the null hypothesis that the intercept is 0.

All *P* values are 2-tailed, and *P* < .05 was considered significant for all analyses. Statistical analyses were performed using SAS version 9.4 (SAS Institute).

## Results

### Study population and reasons for surgical turndown

**Figure 1:**
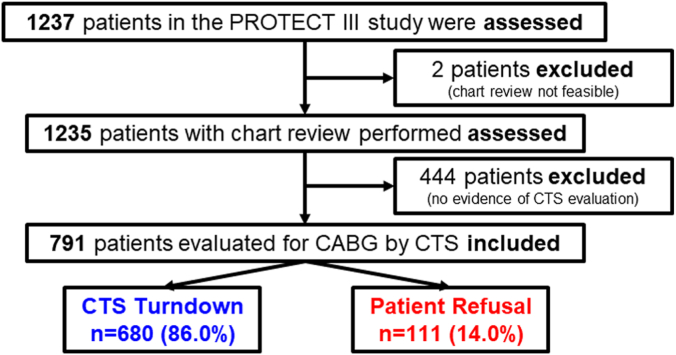
**Study flow chart.** CABG, coronary artery bypass graft; CTS, cardiothoracic surgeon.

Of the 1237 patients enrolled in the cVAD PROTECT III registry, 2 patients who had charts that were not evaluable and another 444 who did not undergo evaluation by a CTS were excluded. The remaining 791 patients who were evaluated by a CTS, but did not undergo surgery due to either surgical turndown (n = 680; 86.0%) or patient declination (n = 111; 14.0%) comprised the final study sample (Figure 1).

**Figure 2:**
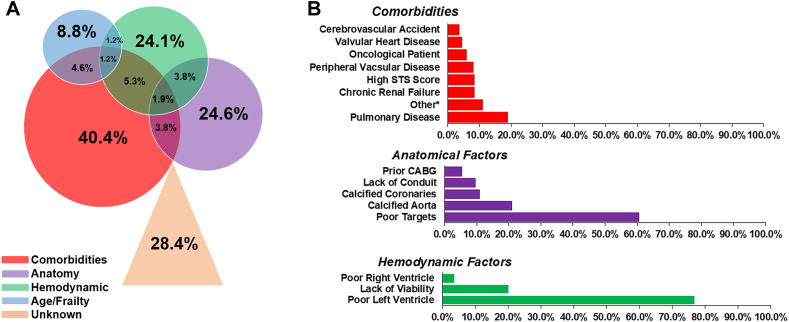
**Etiologies for CABG turndown as evaluated by cardiothoracic surgeons. (A) Venn Diagram of etiologies. (B) Distribution of comorbidities, anatomical factors, and hemodynamic factors.** CABG, coronary artery bypass graft; STS, Society of Thoracic Surgeons.

The reasons for surgical turndown are shown in Figure 2A. Comorbidities were most frequently cited (40.4%), followed by anatomical factors (24.6%), hemodynamic concerns (24.1%), and age/frailty (8.8%). In 28.4% of patients, there was no clearly documented reason for surgical turndown. Categories were not mutually exclusive; the most common combination of reasons for turndown was comorbidities and age (9.3%).

The specific reasons for surgical turndown within each category are shown in Figure 2B. Among patients turned down for comorbidities, pulmonary disease was most commonly cited (19.3%), followed by renal failure (8.4%), high STS risk (8.4%), peripheral vascular disease (8.0%), and cancer (5.5%). Poor target vessel quality was cited as the most common anatomical reason for surgical turndown (61%), followed by calcified aorta (21.0%), calcified coronary target zone (10.8%), absence of bypass conduit (9.6%) and previous CABG (5.4%). Of hemodynamic reasons for turndown, poor LV function was the predominant cause (76.8%), followed by lack of myocardial viability (20.1%) and poor right ventricular function (3.7%).

### Patient characteristics

**Figure 3:**
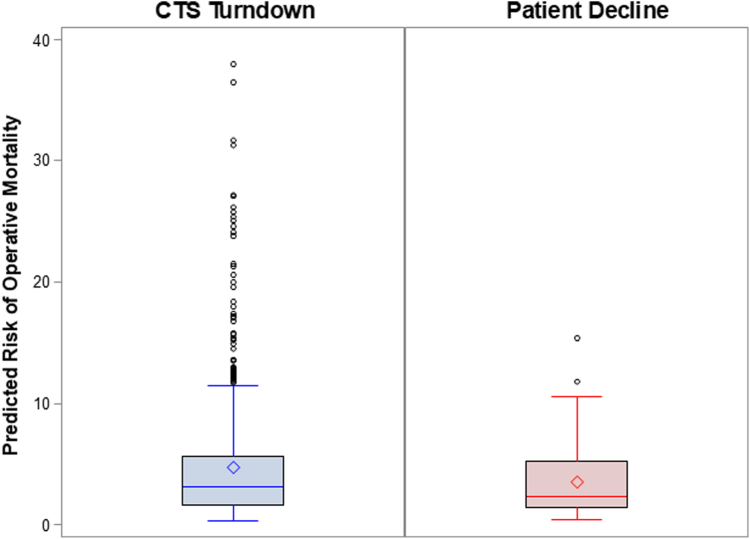
**STS distribution in CTS turndown and patient CABG decline at baseline.** CTS, cardiothoracic surgeon; STS, Society of Thoracic Surgeons.

The baseline characteristics of these patients are summarized in Table 1. When compared with patients who declined CABG, patients who were turned down by a CTS were younger (70.3 ± 11.1 vs 74.4 ± 10.7; *P* = .0004); were less likely to have had previous PCI (31.8% vs 41.8%; *P* = .04); had lower baseline LV ejection fraction (35.2 ± 15.3 vs 38.9 ± 15.3; *P* = .047) and higher prevalence of chronic pulmonary disease (26.0% vs 16.5%; *P* = .03) and chronic kidney disease (33.4% vs 18.9%; *P* = .002); and were less likely to present with a chronic coronary syndrome (30.0% vs 46.1%; *P* = .001). The majority of patients in both groups had left main and 3-vessel diseases. Additionally, there were no significant differences in the number of diseased vessels or the number of treated vessels between groups. The STS score distribution varied between groups. Patients turned down by a CTS had a higher median score and a broader IQR and numerous high-risk outliers at baseline (Figure 3). This was linked to higher STS 30-day predicted risk of mortality in patients who were declined for surgery compared with patients who declined surgery (4.7% vs 3.6%; *P* = .02).

**Table 1:** Baseline demographics and procedural characteristics of patients eligible for CABG.

Characteristics	CTS turndown (n = 680)	Patient decline (n = 111)	Overall *P*
Age, y	70.3 ± 11.1; n = 680	74.4 ± 10.7; n = 111	.0004
Male sex	71.8 (488/680)	73.9 (82/111)	.65
Race			
White or Caucasian	70.1 (477/680)	64.9 (72/111)	.26
Black or African American	11.8 (80/680)	6.3 (7/111)	.09
Asian	2.2 (15/680)	2.7 (3/111)	.74
American Indian or Alaska native	0.6 (4/680)	0 (0/111)	.42
Native Hawaiian/Other Pacific Islander	0 (0/680)	0 (0/111)	NA
Other race	3.4 (23/680)	5.4 (6/111)	.29
Unknown race	11.9 (23/680)	20.7 (23/111)	.01
Body mass index, kg/m^2^	28.8 ± 6.9; n = 678	28.6 ± 5.8; n = 111	.76
Medical history			
History of tobacco use	63.8 (425/666)	60.6 (66/109)	.51
Hypertension	90.5 (612/676)	93.6 (103/110)	.29
Dyslipidemia	79.5 (535/673)	81.7 (89/109)	.60
Diabetes mellitus	59.1 (399/675)	50.5 (56/111)	.09
Peripheral vascular disease	24.1 (161/667)	21.6 (24/111)	.56
Chronic pulmonary disease	26.0 (174/669)	16.5 (18/109)	.03
Stroke/TIA	17.4 (117/671)	16.2 (18/111)	.75
Renal insufficiency	33.4 (224/671)	18.9 (21/111)	.002
eGFR^a^, mL/min/1.73 m^2^	68.4 ± 25.5; n = 552	69.0 ± 23.4; n = 87	.84
On dialysis	28.6 (6/224)	23.8 (5/21)	.64
Anemia	22.2 (133/600)	16.0 (16/100)	.16
Previous PCI	31.8 (212/667)	41.8 (46/110)	.04
Previous CABG	6.4 (43/675)	4.5 (5/111)	.45
Heart failure	59.7 (401/672)	55.5 (61/110)	.40
Left ventricular ejection fraction, %	35.2 ± 15.3; n = 561	38.9 ± 15.3; n = 77	.047
Atrial fibrillation	36.4 (20/55)	60.0 (6/10)	.16
Indication for PCI			
Acute coronary syndrome	34.1 (216/634)	30.4 (31/102)	.47
NSTEMI	79.3 (165/208)	83.3 (25/30)	.61
STEMI	8.7 (18/208)	6.7 (2/30)	.71
Unstable angina	12.0 (25/208)	10.0 (3/30)	.75
Chronic coronary syndrome	30.0 (190/634)	46.1 (47/102)	.001
No. of diseased vessels			.77
1	8.5 (57/672)	7.2 (8/111)	.65
2	26.9 (181/672)	31.5 (35/111)	.32
3	62.9 (423/672)	59.5 (66/111)	.48
>3	1.6 (11/672)	1.8 (2/111)	.90
Left main disease	61.8 (418/676)	70.3 (78/111)	.09
No. of vessels treated			.27
1	24.2 (154/637)	26.0 (27/104)	.69
2	46.6 (297/637)	38.5 (40/104)	.12
3	29.2 (186/637)	35.6 (37/104)	.19
STS risk score (operative mortality)	3.2 [1.7-5.6]; n = 680	2.3 [1.4-5.3]; n = 111	.049

Data are presented as % (n/N), mean ± SD, or median [Q1, Q3], where applicable.

CABG, coronary artery bypass surgery; CTS, cardiothoracic surgeon; eGFR, estimated glomerular filtration rate; NSTEMI, non–ST-elevation myocardial infarction; PCI, percutaneous coronary intervention; STEMI, ST-elevation myocardial infarction; STS, Society of Thoracic Surgeons; TIA, transient ischemic attack.

a eGFR was calculated using 2021 CKD-EPI Creatinine Equation.

### Baseline procedural characteristics and complications

Procedural characteristics, complications, and in-hospital AEs are summarized in Table 2. Patients who were turned down by a CTS had longer PCI procedures (2.2 ± 1.1 vs 1.8 ± 1.0 hours; *P* = .002) and longer hospital length of stay than patients who declined CABG (10.2 ± 21.6 vs 6.5 ± 5.8 days; *P* = .07). Pre-PCI Synergy Between Percutaneous Coronary Intervention with Taxus and Cardiac Surgery (SYNTAX) scores (29.0 ± 12.4 vs 27.9 ± 11.0; *P* = .45) and post-PCI SYNTAX scores (6.8 ± 8.2 vs 5.2 ± 8.8; *P* = .10) were similar between groups. Pre-PCI ischemia jeopardy scores were also similar; however, patients who were turned down by a CTS had higher residual post-PCI ischemia jeopardy scores (2.0 ± 2.1 vs 1.5 ± 2.0; *P* = .02). Coronary atherectomy was used more frequently in patients turned down by CTS (50.9% vs 39.9%; *P* = .03); other procedure usages (eg, optical coherence tomography, intravascular ultrasound, fractional flow reserve, temporary pacer, and aspiration thrombectomy) were similar between groups. While PCI complication rates were similar between groups, post-PCI in-hospital AEs (mainly driven by acute kidney injury, hematoma, and blood transfusion) occurred more frequently in patients who were turned down by a CTS than in those who declined CABG (31.3% vs 21.6%; *P* = .04).

**Table 2:** Procedural characteristics, PCI-related complications and in-hospital adverse events in coronary artery bypass graft–ineligible patients.

Characteristics	CTS turndown (n = 680)	Patient declined (n = 111)	Overall *P*
Duration of index PCI procedure, h	2.2 ± 1.1; n = 633	1.8 ± 1.0; n = 103	.002
Duration of hospitalization, d	7.0 [4.0, 12.0]; n = 673	5.0 [2.0, 10.0]; n = 110	.0003
Duration of Impella support, h	1.69 [1.13, 2.50]; n = 598	1.31 [1.02, 2.10]; n = 92	.007
Pre-PCI and post-PCI SYNTAX and ischemia jeopardy score			
Pre-PCI SYNTAX score	29.0 ± 12.4; n = 471	27.9 ± 11.0; n = 81	.45
Post-PCI SYNTAX score	6.8 ± 8.2; n = 467	5.2 ± 8.8; n = 79	.10
Pre-PCI ischemia jeopardy score	9.3 ± 1.9; n = 544	9.0 ± 1.8; n = 97	.14
Post-PCI ischemia jeopardy score	2.0 ± 2.1; n = 540	1.5 ± 2.0; n = 95	.02
Adjunct therapy and diagnostics used	68.4 (459/671)	71.8 (79/110)	.47
Atherectomy	39.9 (268/671)	50.9 (56/110)	.03
Aspiration thrombectomy	1.0 (7/671)	0 (0/110)	.28
Optical coherence tomography	1.8 (12/671)	0.9 (1/110)	.50
Intravascular ultrasound	47.8 (321/671)	38.2 (42/110)	.06
Fractional flow reserve	2.4 (16/671)	1.8 (2/110)	.71
Temporary pacer	10.9 (73/671)	8.2 (9/110)	.39
PCI-related complications^a^	3.9 (24/621)	5.8 (6/103)	.36
No reflow	0.2 (1/621)	0 (0/103)	.68
Abrupt closure	0.2 (1/621)	1.0 (1/103)	.15
Coronary dissection	0.6 (4/621)	1.0 (1/103)	.71
Distal embolization	0 (0/621)	0 (0/103)	NA
Coronary perforation	1.0 (6/621)	1.9 (2/103)	.38
Arrhythmia	0.3 (2/621)	0 (0/103)	.56
Cardiac arrest	0.3 (2/621)	0 (0/103)	.56
In-hospital adverse events	31.3 (213/680)	21.6 (24/111)	.04
Pericardial effusion requiring pericardiocentesis	1.2 (8/680)	0.9 (1/111)	.80
Cardiac arrest	2.2 (15/680)	0.9 (1/111)	.37
Cardiogenic shock	2.1 (14/680)	1.8 (1/111)	.86
Ventricular arrhythmia	1.9 (13/680)	0.9 (1/111)	.45
AKI (stage 2 or 3)	4.9 (33/680)	4.5 (5/111)	.87
Life-threatening/disabling/major bleeding (BARC scale ≥ 3a)	2.4 (16/680)	4.5 (5/111)	.19
Hematoma	7.6 (52/680)	8.1 (9/111)	.87
Hemolysis	0.9 (6/680)	0 (0/111)	.32
Vascular complication requiring planned surgery	0.9 (6/680)	0.9 (1/111)	.98
Vascular/cardiac structural complication requiring surgery/reintervention	1.2 (8/680)	3.6 (4/111)	.052
Vascular complication without surgery	1.6 (11/680)	0.9 (1/111)	.57
Limb ischemia	1.3 (9/680)	1.8 (2/111)	.69
Anemia requiring transfusion	9.9 (67/680)	6.3 (7/111)	.23
Prolonged intubation (≥48 h)	0.6 (4/680)	0 (0/111)	.42
Neurologic dysfunction (stroke or TIA)	1.3 (9/680)	0 (0/111)	.22

Data are presented as % (n/N) or mean ± SD, where applicable.

AKI, acute kidney injury; BARC, Bleeding Academic Research Consortium; CTS, cardiothoracic surgeon; PCI, percutaneous coronary intervention; SYNTAX, Synergy Between Percutaneous Coronary Intervention with Taxus and Cardiac Surgery; TIA, transient ischemic stroke.

a PCI-related complications are defined as the composite of no reflow, abrupt closure, dissection, distal embolus, and perforation during PCI.

### Major adverse cardiovascular and cerebrovascular events

**Figure 4:**
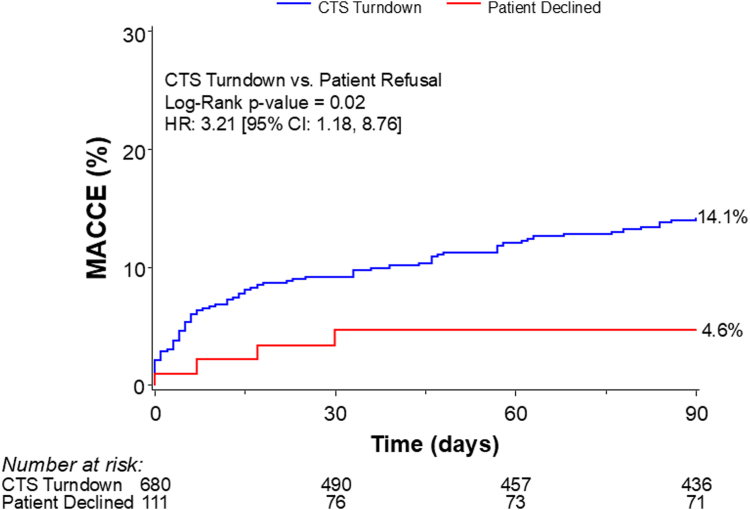
**Kaplan-Meier curves for 30- and 90-day MACCE for CABG candidate patients.** MACCE is defined as the composite of all-cause death, myocardial infarction, stroke/transient ischemic attack, and revascularization. CABG, coronary artery bypass graft; MACCE, major adverse cardiovascular and cerebrovascular event.

At 30 days, patients turned down by a CTS showed numerically higher MACCE rates compared with those who declined surgery and comparable individual components of 30-day MACCE (Table 3). At 90 days, MACCE rates were significantly higher in the CTS turndown group (14.1%) compared with those in both the patient decline group (4.6%) and the overall PROTECT III cohort (12.6%) (Figure 4, Central Illustration). MACCE rates were primarily driven by higher rates of cardiovascular death and myocardial infarction in the CTS turndown group (Table 3).

**Table 3:** Major adverse cardiac and cerebrovascular events at 30 and 90 d and mortality at 1 y.

Outcome	CTS turndown	Patient declined	Overall *P*
30-d MACCE^a^	9.2 (56)	4.6 (4)	.12
Death	8.0 (47)	4.6 (4)	.26
Noncardiovascular	0.9 (5)	0 (0)	.38
Cardiovascular	7.1 (42)	4.6 (4)	.37
Myocardial infarction	2.2 (13)	0 (8)	.16
Stroke/TIA	1.8 (12)	0 (0)	.16
Repeat revascularization	0.4 (2)	0 (0)	.59
90-d MACCE^a^	14.1 (82)	4.6 (4)	.02
Death	12.2 (69)	4.6 (4)	.050
Noncardiovascular	1.3 (7)	0 (0)	.30
Cardiovascular	11.0 (62)	4.6 (4)	.09
Myocardial infarction	4.2 (23)	0 (0)	.059
Stroke/TIA	2.0 (13)	0 (0)	.15
Repeat revascularization	1.6 (8)	0 (0)	.27
1-y mortality	22.3 (119)	11.7 (9)	.03

Event rates are Kaplan-Meier event rates (No. of events) and compared by the log-rank test.

CTS, cardiothoracic surgeon; MACCE, major adverse cardiovascular and cerebrovascular events; TIA, transient ischemic attack.

a MACCE is defined as the composite of all-cause death, myocardial infarction, stroke/TIA, and revascularization. MACCE was adjudicated by CEC; 1-y mortality was site reported.

**Central Illustration:**
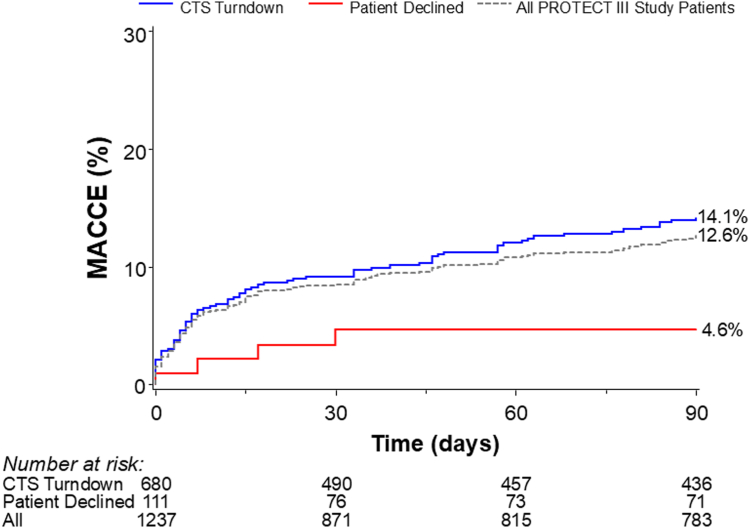
**Kaplan-Meier curves for 90 day MACCE in CTS turndown, CABG patient decline, and all PROTECT III study patients.** CTS, cardiothoracic surgeon; MACCE, major adverse cardiovascular and cerebrovascular event.

### Comparison of observed vs predicted outcomes

**Figure 5:**
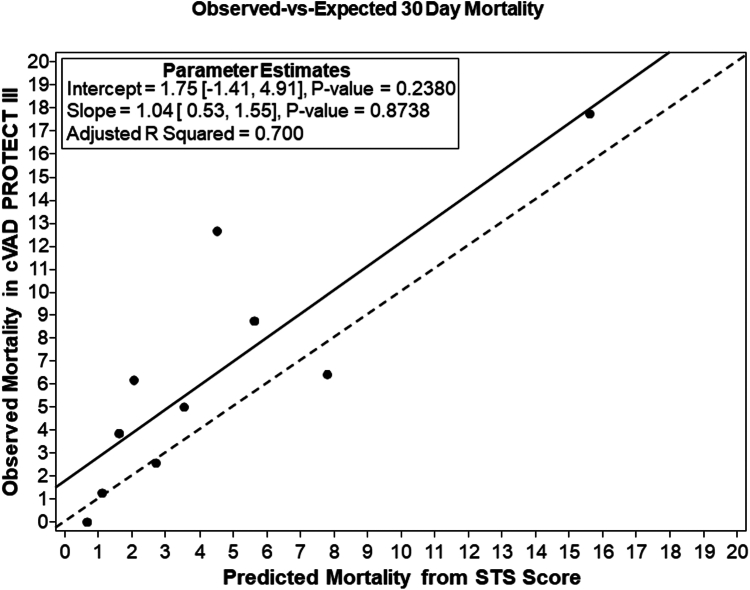
**Relationship between predicted mortality (STS score) and observed mortality at 30 days in patients evaluated for CABG in the cVAD PROTECT III cohort.** CABG, coronary artery bypass graft; STS, Society of Thoracic Surgeons.

**Figure 6:**
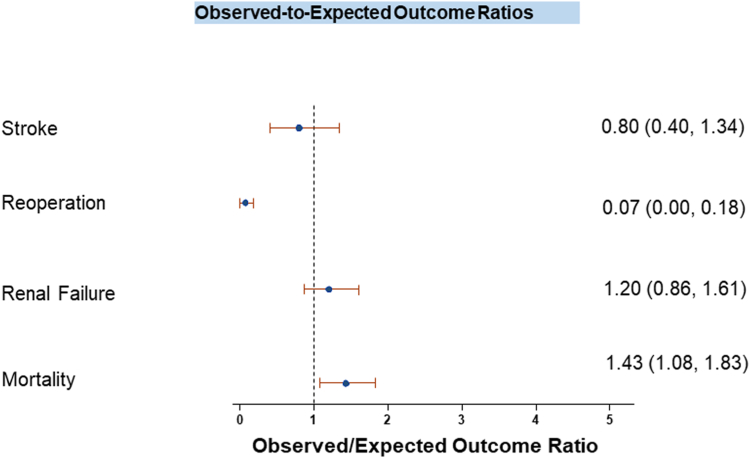
**Observed to expected outcome ratios at 30 days in patients evaluated for CABG in the cVAD PROTECT III cohort.** CABG, coronary artery bypass graft.

The mean STS-predicted 30-day risk of mortality for the entire cohort (n = 791 patients) was 4.52%. The relationship between predicted mortality (based on the STS score) and observed mortality is shown in Figure 5. The line of best fit had a slope of 1.04 (95% CI, 0.53-1.55; *P* = .87), indicating a close match between predicted and observed mortality rates. An intercept of 1.75 (95% CI, −1.41 to 4.91; *P* = .24) showed that the observed mortality was higher than predicted. The adjusted *R*^2^ of 0.700 suggested that 70% of the variance in observed mortality can be explained by the predicted mortality from the STS score, demonstrating reasonably good correlation between these variables. The ratio of observed to expected mortality showed that observed mortality exceeded the predicted rate, with a ratio of 1.4 (95% CI, 1.08-1.83). Similarly, the ratio for acute kidney failure was 1.2 (95% CI, 0.86-1.6), whereas the observed rate of reoperation was lower than expected (Figure 6).

## Discussion

Patients with complex CAD who are anatomical candidates for CABG but are ineligible due to high surgical risk, limited ability to recover, or refusal of surgery pose a significant and increasing challenge in clinical practice and are usually excluded from trials. Our analysis helps identify a critical gap in the literature by studying the short-term outcomes of the largest cohort to date of patients with complex CAD undergoing pLVAD-supported HRPCI, who either declined surgery or they were deemed ineligible for CABG due to advanced age, frailty with a variety of associated comorbidities, and anatomic or hemodynamic factors. We noted the following key findings: (1) patients who were turned down for CABG by CTS were younger but had more comorbidities, higher baseline STS risk, longer PCI procedure times, more in-hospital AEs, and worse 90-day MACCE rates than patients who declined surgery; (2) comorbidities were the most frequent reasons for surgical turndown, followed by anatomical and hemodynamic factors, respectively; and (3) the STS risk score underestimated short-term mortality in this population, highlighting the need for improved risk prediction models.

To our knowledge, this is the first study to compare 2 cohorts of pLVAD-supported-HRPCI patients who were candidates for CABG but did not undergo surgical revascularization due to CTS turndown or patient declination. Although younger in age, patients turned down by surgery had more comorbidities and a higher baseline STS risk score, which correlated with higher 90-day MACCE rates and 1-year all-cause mortality. The reasons for surgical turndown were ascertained, providing further insights. Consistent with previous studies, the most common reasons for surgical turndown were poor target vessels, advanced age, and renal insufficiency in patients with left main or multivessel disease.^16^ The distribution of STS risk scores as noted in Figure 5 indicate that CTS-declined patients tend to be an overall severely ill but heterogeneous population. A higher rate of in-hospital AEs was observed among these patients and a 3-fold increase in 90-day MACCE. These findings are consistent with recent real-world data, which demonstrated a 2-fold increase in mortality for patients deemed surgically ineligible compared with those who refused CABG.^11^ While most patients in this cohort were declined for CABG by a CTS, there are multiple reasons why surgically eligible patients may decline CABG and opt for PCI, even when CABG may offer superior long-term results. These include a preference for less invasive approaches, faster recovery times (particularly for patients with preadmission physical mobility impairments, essential home responsibilities, and desires to avoid postoperative rehabilitation facility or nursing home stays), cultural influences, and levels of health literacy.^17^, ^18^, ^19^ This real-world bias toward PCI has been observed in previous studies, where only 53% of patients with an established indication for CABG undergo CABG.^20^ In the SYNTAX study, 16.4% of patients who were eligible and screened for CABG declined surgical revascularization.^21^ These findings together highlight a pervasive patient preference for PCI despite an evidence base that favors CABG, which compels practitioners to incorporate treatment guidelines, patient age, comorbidities, physical and cognitive function, operative risks, anatomic and hemodynamic factors, and patient values into shared decision making when rendering collaborative heart team revascularization recommendations to high-risk patients with complex CAD.

Our study provided a unique opportunity to evaluate the performance of the STS risk model in predicting 30-day mortality among HRPCI patients supported by temporary pLVAD. Currently, there are no well-established risk prediction models for Impella-supported HRPCI. Although the STS risk prediction model performed well for stroke and renal failure, it performed less well in predicting 30-day mortality. Moreover, although the STS score was validated in the CABG cohort, it appears to underestimate mortality risk in this Impella-supported HRPCI population. The 14% 90-day mortality observed in HRPCI patients turned down by a CTS and a ratio of 1.4 for observed to expected mortality at 30 days, along with higher STS risk score, highlight the extreme risk profile of this cohort. Notably, the STS-predicted 30-day risk of mortality in patients turned down by a CTS was 3.2%, significantly lower than the observed 8% mortality rate. Similar findings were observed when comparing MACCE rates in the CTS turndown group with those of the rest of the PROTECT III cohort (Figure 4). These findings suggest that patients receiving pLVAD support for HRPCI may have a higher-than-estimated short-term mortality risk, which is not fully captured by the STS risk model. One potential explanation is that surgically ineligible patients were not included in the data sets used to develop STS predictive models, limiting their applicability to this population. Additionally, neither the STS nor EuroSCORE II incorporate physical and cognitive deficits affecting frailty, which may further reduce their accuracy in risk stratification for HRPCI patients. Notably, these models were developed without including surgically ineligible patients. It is likely that the factors associated with poor outcomes with CABG also correlate with higher risk in pLVAD-supported HRPCI. However, since surgically ineligible patients were not included in the data sets used to derive the STS models, it is possible that mortality could have been even higher if these patients had undergone surgical revascularization. These findings highlight the need for more accurate risk prediction models for contemporary patients with prohibitive surgical risk undergoing pLVAD-supported HRPCI.

Over time, the risk profiles of patients undergoing CABG have changed, with increasing age and higher burden of comorbidities leading to greater operative risk, and more patients being deemed ineligible due to the risk of perioperative complications.^22^, ^23^, ^24^ However, advancements in PCI techniques, particularly when combined with hemodynamic support, offer an alternative treatment option for these high-risk patients who otherwise may not have been offered revascularization. In appropriately selected patients, this approach can offer a viable revascularization option, improve procedural hemodynamics, and potentially enhance patient quality of life.^13^^,^^25^ Planned use of Impella in this setting may facilitate more complete revascularization and has been shown to reduce AEs and mortality compared with bailout use.^26^ Understanding the relative risks of HRPCI in patients who are anatomical candidates but ineligible for CABG is crucial for case planning and optimal care delivery. Additionally, there is also a growing need for randomized trials to further evaluate the risks vs benefits of planned hemodynamic support in these high-risk cohorts. The PROTECT IV randomized controlled trial is currently ongoing and is expected to provide additional evidence regarding the role of planned hemodynamic support with Impella in HRPCI.

### Limitations

As a single-arm, retrospective observational study, this analysis is subject to bias, and the lack of a control group prevents any conclusions regarding the relative risks and benefits of Impella-supported HRPCI. There was not an equal distribution of sexes in the study population, which should be taken into consideration when interpreting the results. The decision to use an Impella device was left to physician discretion, introducing potential selection bias. Our study does not account for baseline cognitive and physical function, frailty, and other factors that might impact surgical and heart team decision making. Longer follow-up is needed to assess long-term outcomes in this patient cohort.

## Conclusion

This study highlights the complexity of Impella-supported HRPCI in patients who were considered candidates for, but not undergoing, CABG in the PROTECT III trial. Pulmonary disease, poor target vessels, and reduced LV systolic function were the most commonly cited clinical, anatomic, and hemodynamic reasons, respectively, for surgical turndown. Although younger, patients turned down by CTS had a higher burden of comorbidities and experienced worse outcomes compared with those who declined CABG. The STS risk score underestimated short-term mortality**,** emphasizing the need for improved risk prediction models. Although Impella-supported HRPCI offered a viable revascularization option for these high-risk patients, further randomized trials are needed to confirm the risks vs benefits of this strategy.

## Peer review statement

Deputy Editor Suzanne J. Baron, Associate Editor Cindy L. Grines, and Guest Editor Wayne B. Batchelor had no involvement in the peer review of this article and have no access to information regarding its peer review. Full responsibility for the editorial process for this article was delegated to Associate Editor Sandeep Nathan.

## Declaration of competing interest

Yousuf Shah is the founder of Meducator LLC, which facilitated in some programming analyses. David J. Cohen reports institutional research support from Abbott, Edwards Lifesciences, Boston Scientific, Philips, and Zoll Medical and consulting income from Abbott, Edwards Lifesciences, Boston Scientific, Medtronic, and Elixir Medical. Björn Redfors has received consultant fees from Pfizer and Boehringer Ingelheim. Suzanne J. Baron reports receiving consulting fees/speaking honoraria/advisory board membership at Zoll Medical, Edwards Lifesciences, Medtronic; Abiomed; Boston Scientific Corporation, and Picardia and intuitional research support from Boston Scientific Corporation, Abiomed, and Acarix. Mir B. Basir reports consultant fees from Abbott Vascular, Abiomed, Cardiovascular Systems, Chiesi, and Zoll. Aditya S. Bharadwaj has received consultant and speaker fees from Abiomed Inc, Cardiovascular Systems Inc, and Shockwave Medical. Cindy L. Grines reports participation on the advisory boards for Philips and Abiomed. Alejandro Lemor reports speaker fees from Abiomed. Alexander G. Truesdell reports speaking and consulting fees from Abiomed, Getinge, Shockwave, and Zoll. William W. O’Neill reports grant/research support from St. Jude Medical, Edwards Life Sciences, and Biomed; reports consulting fees/honoraria from Medtronic and Abiomed; and is a major stock shareholder/equity in Synecor, Accumed, Neovasc, Tendyne, and Mitral Align. Wayne B. Batchelor has received consulting fees from Boston Scientific, Edwards, Chiesi, and Medtronic. The other authors reported no financial interests.
